# Relacorilant or surgery improved hemostatic markers in Cushing syndrome

**DOI:** 10.1007/s40618-024-02468-2

**Published:** 2024-09-21

**Authors:** C. Simeoli, N. Di Paola, A. Stigliano, P. Lardo, T. Kearney, E. Mezosi, E. Ghigo, R. Giordano, C. N. Mariash, D. M. Donegan, R. A. Feelders, A. L. Hand, K. A. Araque, A. G. Moraitis, R. Pivonello

**Affiliations:** 1 Dipartimento di Medicina Clinica e Chirurgia, Sezione di Endocrinologia, Diabetologia, Andrologia e Nutrizione, Università “Federico II” di Napoli, Naples, Italy; 2 Endocrinology, Sant’Andrea University Hospital, Sapienza University of Rome, Rome, Italy; 3 Salford Royal Foundation Trust, Salford, Manchester, UK; 4 1st Department of Internal Medicine, Clinical Center, University of Pecs, Pecs, Hungary; 5 Division of Endocrinology, Diabetology and Metabolism, University of Turin, Turin, Italy; 6 Department of Biological and Clinical Sciences, University of Turin, Turin, Italy; 7 Indiana University, Indiana University Health, Indianapolis, IN USA; 8 Indiana University School of Medicine, Indianapolis, IN USA; 9 Department of Internal Medicine, Division of Endocrinology, Erasmus Medical Center, Rotterdam, The Netherlands; 10 Corcept Therapeutics Incorporated, Redwood City, CA 94065 USA

**Keywords:** Cushing syndrome, Coagulation factor, Surgery, Relacorilant, Hypercoagulable state

## Abstract

**Purpose:**

Glucocorticoid-mediated hypercoagulability can persist in patients with endogenous Cushing syndrome (CS) after curative surgery and may transiently worsen early postoperatively. These studies aimed to characterize coagulation markers at baseline in patients with CS and the impact of relacorilant or remission post-surgery in an open-label, phase 2 study (NCT02804750) and a retrospective, longitudinal, surgical cohort study.

**Methods:**

In the relacorilant study, 34 patients received relacorilant (100–200 mg/day for up to 12 weeks or 250–400 mg/day for up to 16 weeks) and had postbaseline data. Coagulation markers were assessed before and during treatment. In the surgical study, conducted at “Federico II” University of Naples, Italy, coagulation markers were assessed in 30 patients before surgery and after biochemical remission.

**Results:**

In the relacorilant study, significant mean changes from baseline to last observed visit were reported in factor VIII (− 18.9%, *P* = 0.022), activated partial thromboplastin time (aPTT) (+ 1.5 s, *P* = 0.046), and platelet count (− 68.8*10^9^/L, *P* < 0.0001), whereas von Willebrand factor was unchanged. In the surgical study, the mean time to hemostasis assessment was 6.2 months. Significant mean changes from baseline to hemostasis assessment were reported in factor VIII (− 24.2%, *P* = 0.044), von Willebrand factor (− 20.6%, *P* = 0.018), and aPTT (+ 2.0 s, *P* = 0.031), whereas platelet count was unchanged.

**Conclusions:**

Several coagulation markers improved in patients with CS after 3–4 months of relacorilant treatment and within an average of 6 months after surgery. Relacorilant’s positive effects on coagulation markers support further investigation of its use preoperatively in patients with CS or in patients who are not eligible for surgery.

**Clinical Trial Registration Number:**

NCT0280475 (registration date: 15 June 2016).

## Introduction

Glucocorticoid-induced hypercoagulopathy occurs in patients with endogenous Cushing syndrome (CS) of all etiologies and is associated with thromboembolic complications that contribute to cardiovascular morbidity and mortality [1–7]. Thromboembolic events have been reported in 6% to 20% of patients with CS [4, 8]. According to a systematic meta-analysis, patients with CS had an approximately 18-fold greater risk of venous thromboembolism (VTE) compared to the general population [2]. VTE accounts for approximately 4.4% of all deaths in patients with endogenous CS [9]. The risk of VTE appears to be highest in the postoperative setting, with reported rates of up to 5.6% [2, 3, 10]. Patients with CS have a tenfold increased risk of developing arterial and deep VTE up to 1 year after surgery. The incidence of postoperative thrombosis is comparable to the risk of major orthopedic surgery. There is no correlation between the severity of hypercortisolism and the hemostatic abnormalities [5, 11]. In a systematic meta-analysis including 2,083 patients who underwent transsphenoidal surgery (TSS) or adrenalectomy, 4.8% had a VTE within 30 days of surgery [2].

Several alterations in hemostatic parameters are believed to contribute to the hypercoagulable state in CS. These include: (i) increased synthesis of pro-coagulation factors, particularly factor VIII, fibrinogen, von Willebrand factor, and number of platelets; (ii) activation of the coagulation cascade, which results in a shortening of the activated partial thromboplastin time (aPTT); and (iii) increased fibrinolytic inhibitors (TAFI, alpha 2 antiplasmin). These changes result in impaired fibrinolysis and platelet function [2, 5, 7, 12–17]. Additional comorbidities of CS, such as obesity, uncontrolled hypertension, and diabetes, further contribute to the risk of thromboembolism [18].

Notably, the risk of VTE appears to persist in patients with CS following surgical remission [4, 5, 19, 20]. Evaluations of the effects of surgical treatment for CS on patients’ hemostatic parameters demonstrated that hypercoagulopathy persists and even worsens during the immediate postoperative period and then improves within 6 months to a year following surgery [16, 21–23]. A study by Casonato et al. showed worsening hemostatic parameters in patients with Cushing’s disease (CD) within 1 month after surgery, independently of surgical outcome, with improvements beginning about 3 months after successful surgery and normalization by 12 months [22]. Current CD treatment guidelines include recommendations for anticoagulation therapy for up to 2 weeks before surgery and from 1–2 days up to 3 months following surgery [24]. The transient worsening in hemostasis postsurgery may be due in part to increased inflammation following a decrease in cortisol concentrations (and cortisol’s anti-inflammatory effects) after successful surgery [25]. The inflammatory response after successful surgery may activate the coagulation cascade during the immediate postoperative period, which then normalizes over time.

Evaluations of the effects of pharmacological medical treatment for CS on hemostatic parameters are limited [26, 27]. Relacorilant is a selective glucocorticoid receptor modulator (SGRM) in development for the treatment of endogenous CS. In a phase 2, open-label study in patients with CS (NCT02804750), [28] relacorilant provided clinically meaningful improvements in a number of cortisol-excess-related comorbidities, including hypertension and hyperglycemia, without undesirable antiprogesterone effects or drug-induced hypokalemia.

The aim of the current studies was to characterize baseline coagulation parameters and to evaluate the impact of treatment with relacorilant or surgery on the coagulation state in patients with CS, using data from two sources: i) a prospective, multicenter, open-label, phase 2 study of relacorilant and ii) a retrospective, single-center, longitudinal, surgical cohort study.

## Materials and methods

### Study designs

The phase 2 relacorilant study was a prospective, multicenter, open-label, dose-finding study that evaluated the efficacy and safety of relacorilant in 2 dose groups: a low-dose group (100–200 mg/day for 12 weeks) and a high-dose group (250–400 mg/day for 16 weeks) [28]. The dose in each group was increased by 50 mg/day every 4 weeks. The study was conducted between February 2017 and September 2018 (NCT02804750). A post-hoc analysis examined changes in coagulation markers during relacorilant treatment compared to baseline. The phase 2 study was reviewed and approved by the institutional review board (IRB) at each study center and was conducted in accordance with the World Medical Association, Declaration of Helsinki and the International Council on Harmonisation Good Clinical Practice guidelines. Written informed consent to participate in the relacorilant study was obtained from each patient prior to participation [28].

The surgical study was a retrospective, single-center, longitudinal cohort study conducted in patients with CS at the “Federico II” University of Naples, Italy between 2004 and 2021. The objective was to examine hemostatic parameters before surgery and after remission in a cohort of patients that achieved surgical remission. Per institutional guidelines, as a retrospective chart review, the surgical study did not require IRB approval. However, written informed consent with respect to the confidentiality of data collection according to the Italian privacy policy was obtained and recorded in each medical chart at the time of coagulation marker evaluation.

### Patients

The relacorilant study included adults aged 18–80 years with a diagnosis of endogenous CS requiring medical treatment. In addition to having endogenous CS, patients were required to have hypertension (mean systolic blood pressure of 130–170 mmHg and/or mean diastolic blood pressure of 85–110 mmHg based on 24-h ambulatory blood pressure measurements) and/or impaired glucose tolerance (2-h oral glucose tolerance test [oGTT] result for plasma glucose in the range of 140–200 mg/dL) or type 2 diabetes mellitus (fasting glucose > 126 mg/dL and 2-h oGTT result for plasma glucose ≥ 200 mg/dL at 2 h). Patients were excluded if they had systolic blood pressure > 170 mmHg or diastolic blood pressure > 110 mmHg, hemoglobin A1c (HbA1c) > 12%, uncontrolled hypothyroidism or hyperthyroidism, or uncorrected hypoglycemia < 3.5 mEq/L. Detailed patient eligibility for the relacorilant study has been previously published [28].

The surgical study included adults aged 18–80 years with a diagnosis of endogenous CS treated at “Federico II” University of Naples, Italy. Confirmed diagnosis of endogenous CS in the active phase of disease was based on the presence of average urinary free cortisol (UFC) > upper limit of normal (ULN) (mean of 2–3 measurements performed on different days within 1 week) and/or late-night salivary cortisol > ULN and/or increase in midnight serum cortisol (> 1.8 μg/dL), together with lack of suppression (≥ 1.8 μg/dL) in serum cortisol after low-dose (overnight 1-mg and/or 2-days 2-mg) dexamethasone suppression test and a clinical picture suggestive of CS, such as moon facies, dorsocervical fat pad, facial plethora, increased body weight or central obesity, proximal muscle weakness, low bone mass, psychiatric symptoms, easy bruising, hirsutism, striae rubrae, or acne. The diagnosis of CD was confirmed by the presence of a pituitary macroadenoma or large (maximal diameter ≥ 6 mm) microadenoma, or positive gradient at the inferior petrosal sinus sampling (IPSS), performed any time before surgery. The diagnosis of adrenal CS was confirmed by the presence of low or suppressed adrenocorticotropic hormone (ACTH) concentrations and the evidence of unilateral or bilateral adrenal disease on magnetic resonance imaging or computed tomography (CT) scan. The diagnosis of ectopic CS was confirmed by the presence of normal or high ACTH concentrations, the evidence of a lesion on whole body CT scan, and/or negative gradient on IPSS, and/or negative response to the cortisol releasing hormone/desmopressin test. TSS was performed as first-line treatment for CD. Bilateral adrenalectomy was performed in case of TSS failure for CD or for bilateral adrenal CS. Unilateral adrenalectomy was performed for unilateral adrenal CS. Thoracic surgery was performed for the patient with ectopic CS due to bronchial carcinoid. At the time this analysis was conducted, there were no thromboprophylaxis protocols for CS surgical patients at the study center. Biochemical remission postoperatively was defined by cortisol secretion (serum cortisol < 5 μg/dL at the first hormonal assessment), the need for glucocorticoid replacement therapy for hypocortisolism, or lack of recurrence (defined as normal levels of mean 24-h UFC, late-night salivary cortisol, and serum cortisol after low-dose dexamethasone suppression test [< 1.8 μg/dL] over time, up to the study hemostasis assessment).

### Assessments

In the relacorilant study, coagulation markers, including factors VIII, von Willebrand factor, aPTT, and platelets, were measured before treatment and during treatment at weeks 4, 8, 12, 16 and at last observation. Additional ad-hoc assessments of coagulation factors VIII, IX, and X in patients with abnormal values at baseline were also conducted. Blood and urine samples were collected and analyzed centrally (Q^2^ Solutions Laboratories, Valencia, CA, and Q^2^ Solutions Laboratories, Salisbury, UK, were used for aPTT analysis and complete blood count; Quest Diagnostics Nichol Institute, San Juan Capistrano, CA, was used for hormonal testing, clotting assays, and von Willebrand factor antigen). UFC (normal range, 4–50 μg/24 h [11–138 nmol/d]) was measured by tandem mass spectrometry (MS/MS) [28]. Plasma ACTH (7–10 AM normal range, 6–50 pg/mL [1.3–11.1 pmol/L]) was measured with an immunochemiluminescent assay. Determination of aPTT (normal range, 22–34 s) was performed using the photo-optical clot detection method (Sysmex, TOA Medical Electronics, Kobe, Japan). Coagulation factor activity [VIII (normal range 50%–180%), IX (normal range 60%–160%), and X (normal range 70%–150%)] were assessed using photometric clot detection in platelet-poor plasma. Detection of von Willebrand factor antigen (normal range 50%–217%) in plasma was performed using the immunoturbidimetry method.

In the surgical study, coagulation markers, including factor VIII, von Willebrand factor, aPTT, and platelets were measured before surgery and after remission. Blood and urine samples were collected and analyzed by the local laboratories of “Federico II” University of Naples. UFC [normal range 35–135 μg/24 h (96.6–372.6 nmol/d)] and plasma ACTH [normal range, 10–130 pg/mL (2.2–28.6 pmol/L)] were measured by a chemiluminescence immunoassay. Determination of aPTT (normal range 26–40 s) was performed using coagulative method by Instrumentation Laboratory ACL TOP Family 50 series (Werfen USA, Bedford, MA). Factor VIII activity [normal range, 50%–130%] was assessed using chromogenic coagulative methods. Detection of von Willebrand factor antigen [normal range, 50%–150%] in plasma was performed using immunoturbidimetry methods.

### Statistical analysis

Descriptive statistics [eg, mean ± standard deviation [SD] and/or median (range)] were used to summarize patient baseline demographic, clinical characteristics, and coagulation markers in each study. Wilcoxon signed-rank *P*-values were calculated to assess the mean changes from baseline in each study. Statistical calculations were performed using SAS statistical software version 9.4 or higher (SAS Institute, Cary NC) in the relacorilant study and IBM SPSS software version 21 (IBM, Armonk, NY) in the surgical study. For graphical representation, the median over time is displayed to minimize the effect outliers and smaller sample size have on the displayed data.

## Results

### Patients

Thirty-four patients (mean age 48.2 years, 70.6% female) who received at least one dose of relacorilant and had postbaseline data were included in the relacorilant analysis [28]. Thirty patients (mean age 51.3 years, 80.0% female) were included in the surgical study (Table 1). The majority of patients in the relacorilant and surgical studies had ACTH-dependent CS (79.4% and 73.3% respectively). Of the patients with ACTH-dependent CS, four patients in the relacorilant study and one patient in the surgical study were diagnosed with ectopic ACTH secretion [28]. In the relacorilant study, 2 patients received concomitant anticoagulants [dabigatran (n = 1) and enoxaparin (n = 1)] and eight patients received concomitant antiplatelets [acetylsalicylic acid (n = 6), clopidogrel (n = 1), and salicylic acid (n = 1)] starting before or during relacorilant treatment. No patients in the surgical study received anticoagulants or antiplatelet agents as concomitant medications before or after surgery. Smaller percentages of patients in the relacorilant study had abnormal coagulation marker values at baseline compared with patients in the surgical study (Table 1).

**Table 1:** Demographic and clinical characteristics at baseline for the surgical study and relacorilant study

	Relacorilant study (n = 34)	Surgical study (n = 30)
Age, years (median [range])	46.5 (24, 76)	50.5 (23, 73)
Female, n (%)	24 (70.6)	24 (80.0)
Etiology, n (%)		
ACTH-dependent (pituitary or ectopic)	27 (79.4)	22 (73.3)
ACTH-independent (adrenal)	7 (20.6)	8 (26.7)
24-h UFC, µg/24 h xULN	4.2x	4.5x
Mean ± SD	211.9 ± 234.3^a^	615.6 ± 398.2^b^
ACTH in patients with ACTH-dependent CS (pg/mL, mean ± SD)	66.4 ± 28.6^c^	80.4 ± 45.8^d^
Abnormal coagulation markers at baseline		
Factor VIII, n (%)	12 (35.3)	19 (63.3)
Von Willebrand factor, n (%)	6 (17.6)	11 (45.8)^f^
aPTT, n (%)	2 (6.1)^g^	10 (33.3)
Platelet count, n (%)	4 (11.8)	0^ h^

To convert 24-h UFC from µg/24 h to nmol/day, multiply by 2.76. To convert ACTH from pg/mL to pmol/L, multiply by 0.22

^a^By tandem mass spectrometry. Normal range: 4–50 µg/24 h

^b^By immunoassay. Normal range: 35–135 µg/24 h

^c^By immunochemiluminescent assay. Normal range: 6–50 pg/mL

^d^By immunoassay. Normal range: 10–130 pg/mL

^e^ Biochemical remission postoperatively defined by serum cortisol < 5 μg/dL at the first hormonal assessment, need for glucocorticoid replacement therapy for hypocortisolism, or lack of recurrence (defined as normal levels of mean 24-h UFC, late-night salivary cortisol, and serum cortisol after low-dose dexamethasone suppression test (< 1.8 μg/dL) over time, up to the study hemostasis assessment)

^f^Out of 24 patients

^g^Out of 33 patients

^h^Out of 29 patients

ACTH, adrenocorticotropic hormone; aPTT, activated partial thromboplastin time; UFC, urinary free cortisol

### CS treatment outcomes

Treatment outcomes of the relacorilant study have previously been published [28]. Efficacy was assessed based on changes in hypertension and hyperglycemia, along with improvements in other cortisol-related comorbidities. In the low-dose group, 41.7% of patients with hypertension and 15.4% of patients with hyperglycemia achieved response. In the high-dose group, 63.6% of patients with hypertension and 50% of patients with hyperglycemia achieved response.

In the study cohort of patients who underwent surgery, the mean (SD) time to biochemical remission (defined based on serum cortisol < 5 μg/dL, need for glucocorticoid replacement therapy, or lack of recurrence) was 3.4 (2.4) weeks. Mean 24-h UFC decreased from 615.6 µg/24 h at baseline to 87.4 µg/24 h following surgical remission. In patients with ACTH-dependent CS, mean ACTH decreased from 80.4 pg/mL (17.7 pmol/L) to 20.9 pg/mL (4.6 pmol/L) (Table 1).

### Hemostatic and VTE outcomes

**Fig. 1:**
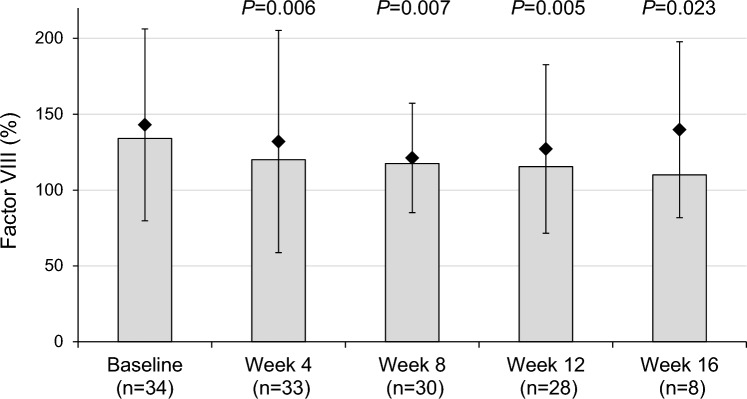
Factor VIII levels during relacorilant treatment. fVIII, factor VIII; SD, standard deviation. Diamonds represent the mean. Error bars represent the SD. Columns represent the median. *P*-values from a Wilcoxon signed-rank test to evaluate if there is a significant change compared to baseline at each visit

In the relacorilant study, significant mean changes from baseline to last observed visit were reported in factor VIII (− 18.9%, *P* = 0.022), aPTT (+ 1.5 s, *P* = 0.046), and platelet count (− 68.8*10^9^/L, *P* < 0.0001), whereas von Willebrand factor was unchanged (Table 2). Changes in factor VIII over time showed decreases from baseline through week 16 (Fig. 1). In patients with abnormal factor VIII at baseline, there was a 54.2% decrease from baseline to last observed visit (*P* = 0.0015). Significant improvements at last observed visit in other coagulation factors, including factors IX (*P* = 0.0293) and X (*P* = 0.0313), were observed in patients with abnormal values at baseline (Table 3).

**Table 2:** Changes in hemostatic parameters in the relacorilant and surgical studies

Relacorilant study	Baseline		Last observed		Change from baseline	*P*-value
	n	Mean ± SD	n	Mean ± SD		
Factor VIII, % [normal range 50–180]	34	143.0 ± 63.2	33	126.4 ± 50.2	-18.9 ± 44.4	0.022
Von Willebrand factor, % [normal range 50–217]	34	145.8 ± 61.4	33	155.0 ± 65.3	+ 6.8 ± 48.6	ns
aPTT, s [normal range 22–34]	33	28.7 ± 10.5	32	28.4 ± 6.9	+ 1.5 ± 6.8	0.046
Platelet count, × 10^9^/L [normal range 130–400]	34	282.7 ± 75.7	34	213.9 ± 68.1	-68.8 ± 61.5	0.0001

Surgical cohort	Baseline		In remission		Change from baseline	*P*-value
	n	Mean ± SD	n	Mean ± SD		
Factor VIII, % [normal range 50–130]	30	161.9 ± 45.8	30	137.7 ± 40.4	-24.2	0.044
Von Willebrand factor, % [normal range 50–150]	24	150.5 ± 61.5	24	129.9 ± 38.4	-20.6	0.018
aPTT, s [normal range 26–40]	30	28.5 ± 4.6	30	30.6 ± 3.4	+ 2.0	0.031
Platelet count, × 10^9^/L [normal range 130–400]	29	269.1 ± 60.5	29	261.1 ± 59.3	-8.0	ns

Wilcoxon signed-rank *P*-values were calculated to assess the mean changes from baseline. In the relacorilant study, data were missing for some patients for baseline or last observed; change from baseline was calculated as last observed value—baseline value, and is missing for patients with either baseline or last observed missing data

aPTT, activated prothrombin time; ns, not significant

**Table 3:** Changes in coagulation factors in patients with abnormal levels at baseline in the relacorilant study

	Baseline		Last observed		Change from baseline	*P*-value
	n	Mean ± SD	n	Mean ± SD		
Factor VIII, % [normal range 50–180]	12	205.0 ± 61.0	12	150.8 ± 60.3	-54.2 ± 40.44	0.0015
Factor IX, % [normal range 60–160]	10	182.7 ± 14.4	10	160.2 ± 31.1	-22.5 ± 28.04	0.0293
Factor X, % [normal range 70–150]	6	167.2 ± 14.8	6	147.0 ± 14.6	-20.2 ± 7.33	0.0313

Data are presented as mean ± SD. SD, standard deviation

In the surgical study, the mean (SD) time to hemostasis assessment was 6.2 (0.8) months. Significant mean changes from baseline to hemostasis assessment were reported in factor VIII (− 24.2%, *P* = 0.044), aPTT (+ 2.0 s, *P* = 0.031), and von Willebrand factor (− 20.6%, *P* = 0.018), whereas platelet count was unchanged (Table 2).

There were no clinically significant VTE events reported in the relacorilant or surgical study.

## Discussion

Data from the current analyses characterize the coagulation profiles of patients with CS participating in the relacorilant and surgical studies. The baseline characteristics of the two patient populations are provided for descriptive purposes and are not intended for a direct comparison. In each study, improvement in several coagulation markers, including factor VIII, in patients with CS during relacorilant treatment and after surgery was observed. Hypercoagulopathy is a known complication in patients with CS of all etiologies, irrespective of disease severity, and contributes to the risk of thrombotic events, including VTE, cardiovascular morbidity, and mortality [1–7, 10, 20]. Glucocorticoids are believed to have a direct effect on the synthesis of various coagulation factors and inhibitors of fibrinolysis [5, 29, 30]. Multiple studies have shown increases in coagulation factors in patients with endogenous CS compared to healthy controls [7, 16, 17, 23, 31]. These increases, mainly in factor VIII and von Willebrand factor, but also in factors IX, X, and XI, and platelet numbers, lead to activation of the coagulation pathway and a shortened aPTT, as well as impaired fibrinolysis and platelet function [7, 16, 17, 23, 31]. Patients with CS also have a significant increase in fast-activating plasminogen activator inhibitor 1, which is the main inhibitor of fibrinolysis, thereby increasing coagulation risk [14, 27, 31]. The presence of inflammation in patients with CS, enhanced oxidative stress, and platelet activation may also have a pathogenic role [13, 32].

Furthermore, higher circulating leukocytes, especially neutrophils, have been reported in patients with CS, and studies also indicate that neutrophils play a prominent role in the occurrence of VTE [33–37]. In the relacorilant study, 7/34 (20.6%) patients had elevated white blood cell (WBC) count at baseline, and 11/33 (33.3%) had elevated absolute neutrophil count at baseline. Mean absolute neutrophil count was in the normal range and remained in the normal range throughout relacorilant treatment. Data on WBC count and neutrophils was not available in the surgical study reported here.

Previous reports from small surgical series have shown that hypercoagulopathy persists short-term after successful surgery and can even increase in the early postoperative period [21, 22]. The time between postoperative remission and normalization of hemostatic parameters varies somewhat among studies [16, 21–23]. The study by Casonato et al. found increases in factor VIII and von Willebrand factor within 1 month after successful surgery, which then decreased over the following months, with normalization by month 12 [22]. Another study by Manetti et al. found significant reductions in several coagulation and fibrinolysis parameters, including von Willebrand factor and plasminogen activator inhibitor-1, 12 months after successful surgery, but not others, such as factor VIII [16]. A study by Ferrante et al. also reported persistent hypercoagulability, measured as endogenous thrombin potential, at 6 months post-surgery, which after 5 years was completely resolved [21], whereas a study from Kastelan et al. observed normalization of several coagulation markers within 6 months of surgical remission [23]. In our analyses of patients with CS, coagulation markers improved after 3–4 months of relacorilant and within an average of 6 months after surgery.

While several coagulation markers improved among patients in both the relacorilant and surgical studies, there were some differences noted between the two studies with regards to effects on von Willebrand factor and platelets. Of note, the percentages of patients with abnormal levels of coagulation markers, including von Willebrand factor, were lower in the relacorilant study than in the surgical study, and the mean baseline levels of the coagulation markers were within the normal ranges in the relacorilant study. The statistically, though not clinically, significant change in platelets in the relacorilant study may have been impacted by the use of anticoagulant and/or antiplatelet medications in several patients in that study. Despite these differences, significant improvements in factor VIII and aPTT occurred in both studies compared to baseline. In addition to its role in platelet adhesion, von Willebrand factor also plays a secondary role in hemostasis via binding and stabilizing factor VIII [38, 39]. The reduction in factor VIII without an accompanying reduction in von Willebrand factor in the relacorilant study may suggest a potential direct effect of relacorilant on the synthesis of factor VIII, resulting in aPTT prolongation. Abnormalities in von Willebrand factor, although frequently observed, are not a constant feature in patients with CS, which may be explained in part by heterogeneity in patient populations and the different assays used [14, 27, 40]. In patients with CS, specific polymorphisms in the promoter haplotype of the von Willebrand gene have been associated with higher levels of von Willebrand factor and VTE risk [40].

Although the present analyses focused on coagulation markers that were assessed in both the surgical and relacorilant study populations, assessments of additional coagulation markers (factors IX and X) conducted in a subset of patients with abnormal levels at baseline also showed improvements following relacorilant treatment. The relacorilant data also demonstrated decreases in median factor VIII throughout each 4-week assessment interval. The previously observed transient increases in factor VIII one month after surgery that were observed by Casonato et al. [22] were not observed in relacorilant-treated patients. In the surgical study, measurement of coagulation markers did not occur immediately following surgery, and thus we are not able to confirm whether coagulation markers worsened initially, similar to the study by Casonato et al.

Similar restorative effects on coagulation factors have not been observed with several other medical agents for CS and CD despite normalization of cortisol concentrations, [26, 27] and the effect of medical pretreatment on VTE outcomes has been mixed [4, 41]. Short-term (80 days), stepwise medical treatment with pasireotide, cabergoline, and ketoconazole, for example, did not lead to a reversal of the hypercoagulable state in patients with CD [27]. Likewise, no significant improvements in coagulation markers were seen in patients with CD after 6–12 months of treatment with pasireotide [26]. A phase 3 study investigating the efficacy and safety of relacorilant in patients with hypercortisolism due to adrenal adenoma or hyperplasia (GRADIENT, NCT04308590) and an open-label extension study of relacorilant in patients with CS (NCT03604198) will include assessment of relacorilant’s impact on coagulation factors. Should data from these analyses support our present findings, pretreatment with relacorilant (once approved) may be beneficial in patients, particularly those at a high risk of VTE, by potentially eliminating the postoperative rise in coagulation factors that can occur following surgery. The data also suggest an additional potential treatment benefit of relacorilant in patients with CS who are not surgical candidates. Whether the improvement in coagulation profile will lead to a reduced risk of VTE remains to be determined. While there were no clinically significant VTE events reported in either the relacorilant or surgical study, this may be due in part to the lack of prospective monitoring for VTEs. Some patients in the relacorilant study also received anticoagulants. This would not have affected the assessment of coagulation markers but could have affected the occurrence of VTEs. Currently, there are no standard practices for thromboprophylaxis in patients with CS. Prophylactic anticoagulation should be considered in patients that are at increased risk of VTEs [24]. While the optimal duration of preoperative and postoperative anticoagulation remains to be determined, experts have recommended prolonged postoperative anticoagulation durations of up to 2–3 months [24].

Limitations of the presented studies include that the studies were designed independently and not with the objective of allowing cross-study comparison of coagulation parameters; that the surgical study was retrospective in nature; that some patients in the relacorilant study received concomitant anticoagulants or antiplatelets before or during the study; the lack of immediate postoperative hemostatic assessment in the surgical study; the post-hoc nature of the relacorilant analyses; the lack of a placebo control group in both studies; and the small number of patients. These analyses focused on coagulation markers that were measured in both studies. As a result, assessments of fibrinolysis or WBC count/neutrophils, which would also be helpful in understanding the impacts of medical treatment and surgery on the coagulation state, were not reported. Gender specific differences could also not be assessed due to the limited number of patients in both studies.

In conclusion, VTE risk due to hypercoagulopathy is a significant concern for patients with CS. These studies showed improvement in several coagulation markers in patients with CS after 3–4 months of relacorilant treatment and within an average of 6 months after surgery. In both studies, factor VIII levels and aPTT improved significantly following treatment. There were no transient increases in factor VIII levels with relacorilant observed during the study. Further analysis of relacorilant’s effect on CS-mediated hypercoagulopathy is ongoing.

## Data Availability

Some or all datasets generated during and/or analyzed during the current study are not publicly available but are available from the corresponding author on reasonable request.
